# Is overexpression of HER-2 a predictor of prognosis in colorectal cancer?

**DOI:** 10.1186/1471-2407-9-1

**Published:** 2009-01-01

**Authors:** Dara O Kavanagh, Gillian Chambers, Liam O' Grady, Kevin M Barry, Ronan P Waldron, Fadel Bennani, Paul W Eustace, Iqdam Tobbia

**Affiliations:** 1 Department of Surgery, Mayo General Hospital, Castlebar, Co Mayo, Ireland; 2 Department of Histopathology, Mayo General Hospital, Castlebar, Co Mayo, Ireland

## Abstract

**Background:**

The development of novel chemotherapeutic agents in colorectal cancer has improved survival. Following initial response to chemotherapeutic strategies many patients develop refractory disease. This poses a significant challenge common to many cancer subtypes. Newer agents such as Bevacizumab have successfully targeted the tyrosine kinase receptor epidermal growth factor receptor in metastatic colorectal cancer. Human epidermal growth factor receptor-2 is another member of the tyrosine kinase receptor family which has been successfully targeted in breast cancer. This may play a role in colorectal cancer. We conducted a clinicopathological study to determine if overexpression of human epidermal growth factor receptor-2 is a predictor of outcome in a cohort of patients with colorectal cancer.

**Methods:**

Clinicopathological data and paraffin-embedded specimens were collected on 132 consecutive patients who underwent colorectal resections over a 24-month period at Mayo General Hospital. Twenty-six contained non-malignant disease. Her-2/neu protein overexpression was detected using immunohistochemistry (IHC). The HER-2 4B5 Ventana monoclonal antibody was used. Fluorescent insitu hybridisation (FISH) was performed using INFORM HER-2/Neu Plus. Results were correlated with established clinical and pathological predictors of outcome including TNM stage. Statistical analysis was performed using SPSS version 11.5.

**Results:**

114 were HER-2/Neu negative using IHC, 7 showed barely perceptible positivity (1+), 9 showed moderate staining (2+) and 2 were strongly positive (3+). There was no correlation with gender, age, grade, Dukes' stage, TNM stage, time to recurrence and 5-year survival (p > 0.05). FISH was applied to all 2+ and 3+ cases as well as some negative cases selected at random. Three were amplified (2 were 3+ and 1 was 2+). Similarly, HER-2 gene overexpression did not correlate with established prognostic indicators.

**Conclusion:**

HER-2 protein is over expressed in 11% of colorectal cancer patients. The gene encoding HER-2 is amplified in 3% of cases. Overexpression of HER-2 is not a predictor of outcome. However, patients who over express HER-2 may respond to Herceptin therapy.

## Background

Colorectal cancer is the second commonest cause of cancer-related death in the United States and the Western World [1,2]. The 5-year relative survival rate is approximately 45% demonstrating an improvement from 30 years ago when the survival rate was 30%. Modifications in adjuvant therapy have been central to this observed improvement. Cytotoxic agents such as irinotecan and oxaliplatin have improved survival while the development of monoclonal antibodies against growth factor receptors has augmented their effects. A significant improvement in the median overall survival in patients with metastatic colorectal cancer was achieved when Bevacizumab (a monoclonal antibody directed against the tyrosine kinase receptor, VEGF) was added to a chemotherapy regimen of oxaliplatin and 5-fluorouracil (P = 0.0024) [3].

The human epidermal growth factor receptor-2 (HER-2) is another member of the tyrosine kinase receptor superfamily. HER-2 protein is also known as c-erb-2 or *neu*. Slamon et al demonstrated that this is over expressed in 30% of patients with breast cancer [4]. Trastuzumab (a monoclonal antibody directed against HER-2) has impacted upon survival in approximately 20% of patients who over express HER-2 and is now established therapy in the treatment of metastatic breast cancer [5]. Other members of the family include HER-1, HER-3 and HER-4. They all possess an extracellular ligand binding domain, a membrane spanning region and a cytoplasmic domain with tyrosine kinase activity. It is understood that HER-2 heterodimerises with other HER receptors unlike the other family receptors which possess a direct binding ligand. In tumour model systems overexpression of the HER-2 gene correlates with mitogenesis, malignant transformation, increased cell motility, invasion and metastasis [6]. Overexpression of HER-2/neu in breast cancer is a useful marker of outcome. It correlates with a poor prognosis. Moreover, it is used to predict patient response to adjuvant chemotherapy and endocrine therapy and to select patients for immunotherapy with targeted monoclonal antibody therapy [7,8]. Intuitively, patients who over express HER-2 should respond to Trastuzumab (Herceptin) therapy independent of the tissue of origin of the cancer. Herceptin has been shown to inhibit colony formation of the HCA-colon cancer cell line and HCA-7 tumour xenografts [9].

Several studies have reported a variety of protein and gene expression levels in colorectal cancer. Similarly there is a wide variability in the published literature in relation to survival benefit. This variety is predominantly due to a lack of standardisation of detection methods. Some studies have demonstrated up to 70% overexpression of cytoplasmic HER-2 [10,11]. Cytoplasmic HER-2 is incapable of transmitting the strong mitogenic signal via heterodimerisation of other members of the EGFR family. Others have reported membranous overexpression in 50% of patients with colorectal cancer [12]. The current study employed the monoclonal antibody 4B5 which is used to determine suitability for Herceptin therapy in breast cancer in our unit. We employed a FISH technique validated for therapeutic use in breast cancer. The aim of the current study was to establish the presence of membranous HER-2 protein overexpression in colorectal cancer, determine if there is gene amplification and establish whether overexpression of HER-2 is a predictor of outcome.

## Methods

A total of 132 patients were included for analysis. These represented consecutive patients who underwent colorectal resections at Mayo General Hospital from January 1^st^, 1998 to December 31^st^, 1999. The study was approved by the institutions' ethics committee. The study group consisted of 48 females and 84 males. The mean age was 67 years (range = 29 – 89 years). None of the patients had undergone preoperative chemotherapy or radiotherapy. The medical records of these patients were reviewed specifically regarding patient demographics, therapeutic strategies, pathological data and survival. Patients were followed up in accordance with international guidelines. Pathological subtypes included (number): adenomatous polyp (10), diverticulosis (7), IBD (5), ischaemia (2), Carcinoid (1), angiodysplasia (1) and colorectal cancer (106). Each specimen was formalin fixed and embedded in paraffin. Prior to inclusion each slide was verified by a pathologist.

### Immunohistochemistry

Staining for HER-2/neu protein was performed on 5 μm (micro-meter) thick slides using the Ventana PATHWAY HER-2 (4B5) mouse monoclonal antibody (Ventana Medical Systems, Tuscon, USA). There is significant correlation of staining between the CB11 antibody which is FDA approved and the HerceptTest. In our institution the 4B5 monoclonal antibody is utilised which is equally valid and used to determine suitability for herceptin therapy in breast cancer. Following deparaffinisation, antigen retrieval and incubation with blocking agent the 4B5 monoclonal antibody directed against HER-2/neu was incubated using an automated slide staining device. At the end of each incubation step, the Ventana automated slide stainer washed the sections and applied a cover slip to minimise evaporation of aqueous reagent. The primary antibody was then localised by a biotin-conjugated secondary antibody formulation that recognises mouse immunoglobulins. The specific antibody-secondary antibody-avidin/streptavidin-enzyme complex was then visualised with a diaminobenzidine stain. They were subsequently counterstained with haematoxylin, dehydrated and cover slips were mounted. Slides were viewed by two independent pathologists blinded to each others findings. Negative controls were created by the omission of primary antibody and replacement with phosphate buffered saline (PBS). Invasive breast cancer specimens were utilised as positive controls. Slides were scored using a four-tiered scoring system according to manufacturer's guidelines. Score 0 is defined as no staining or membranous staining in < 10% of tumour cells. Score 1+ is defined as faint membrane staining in > 10% of tumour cells. Score 2+ is defined as weak to moderate staining in > 10% of tumour cells and a score of 3+ is defined as strong staining of the entire membrane in > 10% of tumour cells. A score of 0 or 1+ was considered negative while a score of 2+ or 3+ was considered positive. Cytoplasmic staining may have been present but was not included in the determination of positivity.

Fluorescent *insitu *hybridisation (FISH) was applied to all 2+ and 3+ cases as well as a random selection of negative cases. This was conducted using the INFORM HER-2/neu plus probe kit. This technique utilises two fluorescent radiolabelled probes: LSI HER-2 (specific to the HER-2 gene locus) and CEP 17 (specific to the alpha satellite DNA sequence at the centromeric region of chromosome 17). The former stains orange while the latter stains green. The slides were evaluated for the HER-2 gene using a fluorescent microscope where a cell is considered to show amplification if > 4 signals of HER-2/neu is detected.

Statistical analysis was performed using SPSS version 11.5. HER-2 protein and gene expression were correlated with clinicopathological parameters and 5-year cancer-related survival using chi-squared testing. Patients who died within 30 days of surgery were excluded from the 5-year cancer-related survival. Patients were followed up for a median of 48.5 months (Range = 1–121 months). For all tests, P < 0.05 was considered to be statistically significant.

## Results

One hundred and thirty two consecutive patients were included in the study. Twenty six of these had non-malignant disease.

### Immunhistochemical detection of HER-2/neu overexpression

**Figure 1:**
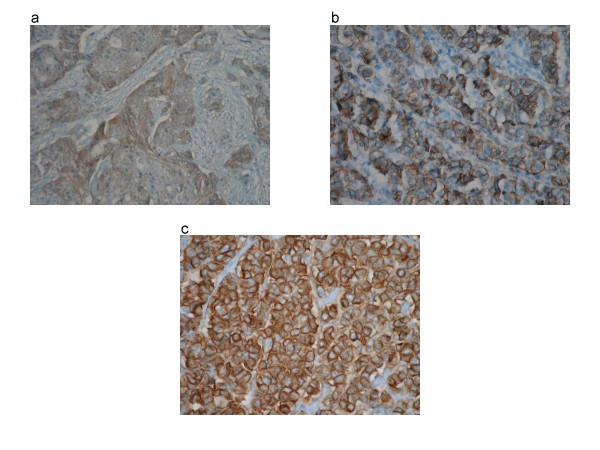
**Immunohistochemical staining for HER-2/neu (Haematoxylin & eosin, magnification × 200)**. Breast cancer cells were used as a positive control (not shown). a.) Faint membrane staining is shown in > 10% of tumour cells (1+). b.) Weak to moderate staining of the entire membrane (2+). c.) Strong staining of the entire membrane in > 10%.

HER-2/neu overexpression was not detected in benign tissues. Ten percent of patients exhibited a score of 2+ or 3+ and were considered positive as shown in Table 1/Figure 1. Positive staining was found in 2 (7%) of the 27 rectal carcinomas and 9 (11%) of the 79 colon cancers. The interobserver agreement was 0.91 as illustrated in Table 2.

**Table 1:** Overexpression of HER-2/neu gene and protein in colorectal cancer

Immunohistochemical score	n (%)	FISH amplification	n (%)
0	0 (0)	> 4 signals/cell	0
1+	7 (7)	> 4 signals/cell	0
2+	9 (8)	> 4 signals/cell	1
3+	2 (2)	> 4 signals/cell	2 (2)

HER-2 protein was not over expressed in non-malignant colorectal specimens. The HER-2/neu gene was not over amplified in selected non-malignant specimens.

**Table 2:** Calculation of the interobserver agreement (kappa coefficient)

			Pathologist 2			Total
			1+	2+	3+	1.00
Pathologist 1	1+	Count (n)	7	1	0	8
		Pathologist 1 (%)	87.5	12.5	0	100
		Pathologist 2 (%)	100	11.1	0	44.4
		% of Total	38.9	5.6	0	44.4
						
	2+	Count (n)	0	8	0	8
		Pathologist 1 (%)	0	100	0	100
		Pathologist 2 (%)	0	88	0	44.4
		% of Total	0	44	0	44.4
						
	3+	Count (n)	0	0	2	2
		Pathologist 1 (%)	0	0	100	100
		Pathologist 2 (%)	0	0	100	11.1
		% of Total	0	0	11.1	11.1
						
	Total	Count (n)	7	9	2	18
		Pathologist 1 (%)	38.9	50	11.1	100
		Pathologist 2 (%)	100	100	100	100
		% of Total	38.9	50	11.1	100

**Symmetric measures**						

	Value	Standard error (a)	Approximate T (b)	Approximate significance		

Kappa coefficient	0.906	0.092	4.894	0.00		
Number of valid cases (n)	18					

a Not assuming the null hypothesis.

b Using the asymptotic standard error assuming the null hypothesis.

The clinical and pathological features of these patients are presented in Table 3. Tumours that over expressed HER-2 were predominantly poorly differentiated and Dukes C. This correlation was not significant (p = 0.72). There was no correlation between HER-2/neu overexpression and age, gender, lymphovascular invasion, TNM stage, perineural invasion or tumour size.

**Table 3:** Clinicopathological features of patients with colorectal cancer (n = 106)

Variable	
Gender (number (%))	
Male	71 (67)
Female	35 (33)
	
Age (Range in years)	67 (29 _89)
	
Dukes stage (number (%))	
A	14 (13)
B	21 (20)
C	55 (52)
D	16 (15)
	
Histological grade (number (%))	
Well	14 (13)
Moderate	65 (61)
Poor	27 (26)
	
Site of tumour (number (%))	
Right colon	18 (17)
Transverse colon	6 (6)
Left colon	55 (52)
Rectum	27 (25)
	
5-year survival (%)	48
Cancer specific 5-year survival (%)	51

### HER-2/neu gene amplification

**Figure 2:**
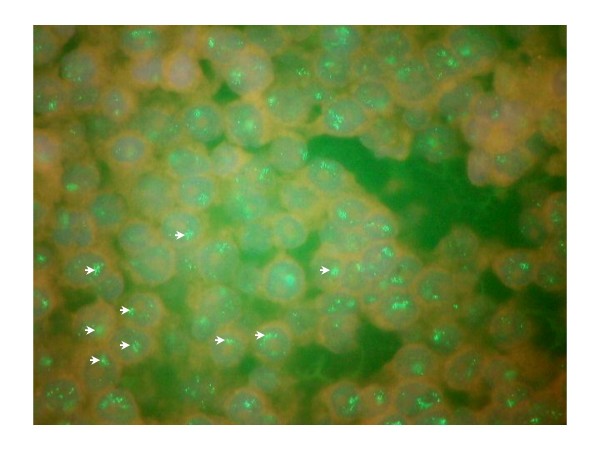
**Fluorescence in situ hybridisation of HER-2 gene amplification**. Amplified HER-2/neu gene forms multiple scattered signals as illustrated with the white arrows.

FISH analysis demonstrated gene amplification in 2 rectal (both 3+) and one colon (2+) cancer (Table 1 & Figure 2). In randomly selected tumours which demonstrated negative staining for HER-2 (0+ and 1+) none revealed amplification of the gene. There was no correlation between gene expression and clinical or pathological parameters.

### Correlation with survival

**Figure 3:**
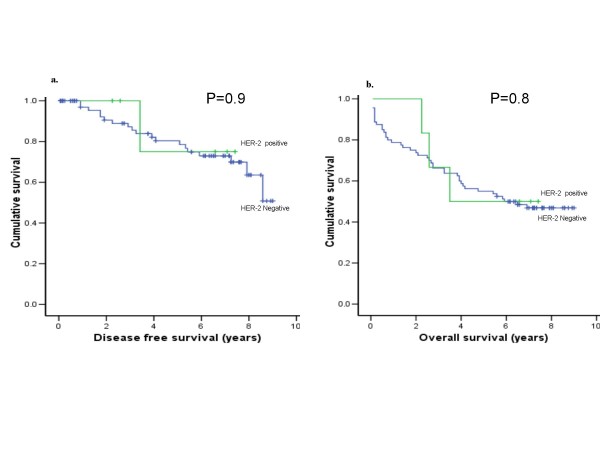
**Kaplan-Meier plot for: a.) disease-free and b.) overall survival in 106 colorectal cancer patients comparing HER-2 positive and HER-2 negative patients**. There was no significant correlation with both parameters and HER-2 overexpression.

This was carried out on patients with cancer-related death who died a minimum of 30 days following resection. The 5-year survival for well, moderately and poorly differentiated cancer was 78%, 58% and 42% respectively. The 5-year survival by Dukes' stage for A, B, C, D were 86%, 78%, 53% and 13% respectively. There was no correlation between HER-2/neu protein overexpression and disease-free or overall survival as illustrated in Figure 3. (p = 0.9: p = 0.8 respectively)

## Discussion

EGFR and HER-2/neu are therapeutic targets in a variety of malignancies due to their overexpression. These can be targeted utilising antibodies directed against the extracellular domains or tyrosine kinase inhibitors. These strategies have been successful in the area of breast cancer and EGFR has been effective in the treatment of metastatic colorectal cancer. Not all patients who overexpress HER-2/neu respond to treatment with Herceptin (25%). The published data report varying rates of HER-2 overexpression in colorectal cancer. The current study was undertaken with a view to establishing a potential role for Herceptin therapy in colorectal cancer by utilising an internationally validated detection method.

Our data demonstrates that HER-2 protein is overexpressed in 11% of cases while the gene is amplified in 3%. While this did not correlate with prognostic indices or survival all cases were poorly differentiated Dukes' C cancers. A lack of consistency between the two techniques has been previously described [12]. This may be interpreted by the hypothesis that protein expression is achieved by transcriptional activation of genes other than the HER-2 gene. Both techniques are used in determining treatment response in breast cancer provided the technique is validated. The wide range of protein (0–70%) and gene (0–30%) expression in colorectal cancer is largely due to the lack of standardisation of the detection methods [13-18]. High protein rates are often reported due to detectable cytoplasmic HER-2 which is not targeted by Herceptin. Immunohistochemistry is relatively inexpensive, widely available, easy to preserve, less time consuming and requires a routine microscope. Assessment by two pathologist with minimal interobserver variability improves the accuracy. The variability in antigen retrieval, antibody type and inconsistent storage of paraffin embedded tissues limits it accuracy. FISH uses a more objective scoring system, is automated and has a sensitivity of 96% and a specificity of 100% [19]. However, it is expensive, time consuming, requires a fluorescent microscope and it is difficult to distinguish tumor cells from normal stromal cells. Emerging results suggests that FISH is more accurate than immunohistochemistry in predicting patient outcome and response to herceptin [20].

We have not demonstrated that overexpression of HER-2 predicts survival however we have shown that it tends to appear in a more aggressive phenotype. Lazaris et al identified a 36% expression rate and a role as an predictor of poor outcome [21]. Similarly Park et al reported a 47% protein expression rate using a polyclonal antibody (Zymed laboratories, South San Francisco, USA) and correlated overexpression with a higher incidence of postoperative recurrence [12]. Conversely, Schuell et al demonstrated an overexpression rate of 4% using the validated HercepTest. This did not correlate with survival [22,23]. The HER-2 overexpression rate of 11% demonstrated in the current study may approach significance in relation to poor prognosis in a larger patient cohort. The methodology utilised is internationally validated in selecting patients who undergo treatment with Herceptin for metastatic breast cancer.

A phase II trial in conjunction with the National Cancer Institute demonstrated low levels of HER-2 overexpression (8%) however when these patients with metastatic colorectal cancer were treated with Herceptin in combination with irinotecan 5 of 7 patients who overexpressed it responded to therapy [24]. Under the current staging system within a specific stage a variable spectrum of outcome exists (> 25% in stage II disease). The development of gene signatures may unfold subsets within a given stage who have a worse prognosis and therefore require different adjuvant therapies [25]. One of these genes may be the HER-2/neu gene.

## Conclusion

We have not demonstrated compelling evidence supporting a potential role for Herceptin in colorectal carcinoma as shown in breast cancer. Similarly we have not revealed that it is an important prognostic indicator. Some patients who overexpress HER-2 (11%) will respond to Herceptin. The evolution of signature genes in colorectal cancer and molecular profiling may facilitate identification of the small subset of patients overexpressing HER-2 who will have a favourable response to Herceptin therapy.

## Competing interests

The authors declare that they have no competing interests.

## Authors' contributions

DK designed and conducted the study. He prepared the manuscript and did the statistical analysis. GC collected the clinicopathological data. LOG performed the laboratory work. KB, RW and PW prepared and corrected the manuscript. FB and IB reviewed the slides, prepared the images and reviewed the manuscript.

## Pre-publication history

The pre-publication history for this paper can be accessed here:

http://www.biomedcentral.com/1471-2407/9/1/prepub
